# Mitral Valve Repair in Advanced Age Groups: Does Cardiac Age Differ from Chronological Age?

**DOI:** 10.3390/jcm12113790

**Published:** 2023-05-31

**Authors:** Roya Ostovar, Filip Schröter, Martin Hartrumpf, Ralf-Uwe Kuehnel, Dunja Bruch, Farnoosh Motazedian, Johannes Maximilian Albes

**Affiliations:** Department of Cardiovascular Surgery, Heart Center Brandenburg, Faculty of Health Sciences, University Hospital Brandenburg Medical School “Theodor Fontane”, 16321 Bernau, Germany; martin.hartrumpf@immanuelalbertinen.de (M.H.); ralf-uwe.kuehnel@immanuelalbertinen.de (R.-U.K.); dunja.bruch@immanuelalbertinen.de (D.B.); farmotazedian@yahoo.com (F.M.); johannes.albes@immanuelalbertinen.de (J.M.A.)

**Keywords:** cardiac aging, cardiac surgery, outcome, aging, mitral valve surgery, mortality

## Abstract

Objective: Advanced age is a risk factor in cardiac surgery contributing significantly to a worse outcome. The reasons are frailty and multimorbidity. In this study, we asked: Is there an aging of the heart which differs from chronological age? Methods: Propensity score matching was performed between 115 seniors ≥ 80 years and 345 juniors < 80 years. After the patients were found to be comparable in terms of cardiac and noncardiac disease and risk profiles, they were further analyzed for cardiac parameters. In addition, the seniors and juniors were compared in terms of cardiac health and postoperative outcome. Furthermore, the patients were subdivided into several age groups (<60 years, 60–69 years, 70–79 years, and >80 years) and compared regarding outcome. Results: The seniors demonstrated significantly lower tricuspid annular plane systolic excursion (TAPSE), significantly more frequent diastolic dysfunction, significantly higher plasma levels of NT-proBNP, and significantly larger left ventricular end-diastolic and end-systolic diameters and left atrial diameters (*p* < 0.001, respectively). Furthermore, in-hospital mortality and most postoperative complications were significantly higher in seniors compared with juniors. While old cardiac healthy patients showed better outcome than old cardiac aged patients, the outcome from young cardiac aged patients was better than old cardiac aged patients. The outcome and survival deteriorated with increasing life decades. Conclusions: The elderly suffer significantly more from cardiac deterioration, i.e., cardiac aging, and show higher multimorbidity. Mortality risk is significantly higher and they suffer more often from a complicated postoperative course compared to younger patients. Further approaches to prevention and treatment of cardiac aging are needed to address the needs of an aging society.

## 1. Introduction

Cardiac disease is one of the most common causes of morbidity and mortality in western countries with aging societies [1,2,3]. Age is generally widely acknowledged as a risk factor for cardiac disease [4,5,6]. However, there is much evidence that cardiac deterioration during aging can be triggered, or accelerated, by various endogenous or exogenous factors [7,8]. For that, the term “cardiac aging” is widely used. “Cardiac age” or “cardiac aging” is still elusive and requires a more precise definition. It is undisputed that “cardiac age” can be different from biological age. However, both sides of the coin must be considered. Having a “younger” heart, i.e., having no or only moderate age-related changes than would be expected chronologically, would correspond to a “favorable cardiac age”. Conversely, having an “older” heart would be an “unfavorable cardiac age”. All cells age, and cardiomyocytes are no exception. It might, therefore, be consistent to correlate age-related physiological changes with defined cellular changes.

In an aging population, not only is there an increase in the number of cardiac diseases and multimorbidity requiring intervention, but also the treatment is more demanding and the postoperative course is often more complex.

The proportion of patients older than 80 years in cardiac surgery has increased over the past 10 years from 13.8% in 2012 to 20.7% in 2021 [9,10]. The cardiac diseases requiring surgery can be very heterogeneous, e.g., from an isolated disease with normal myocardial function to multi-valvular disease and biventricular dysfunction. For example, an isolated disease with an otherwise mostly healthy myocardium and no relevant co-morbidities, presented early enough for surgery, is less problematic even in advanced age. In other words, the challenge in treating the elderly patient lies in often advanced multimorbidity as well as concomitant cardiac disease [11].

Of course, there are always counterexamples in daily clinical practice, such as elderly, spry patients with an uncomplicated course and young, multimorbid patients with a complex course.

Nonetheless, it is already evident that myocardial remodeling with reduced systolic and diastolic dysfunction is related to age [12]. Thus, these changes can be addressed as markers of “cardiac aging” [13]. 

In recent years, there have been an increasing number of experimental studies and animal models dealing with cardiac aging, risk factors and possible therapeutic approaches [14].

The main known clinical factors include hypertension, diabetes mellitus, hyperlipoproteinemia, smoking, obesity and hyperuricemia, the main known cellular factors are oxidative stress, and mitochondrial and lysosomal dysfunctions. These known clinical risk factors can be confirmed from clinical experience in the majority of cardiac surgery patients. 

Accordingly, efforts have been made for years to identify cardiac surgical risk factors and to map them in risk score systems, such as STS score or EuroSCORE, in order to assess the mortality risk in the context of the preoperative setting. For example, not only age but also particular cardiac parameters, such as left ventricular ejection fraction, are addressed in the EuroSCORE system. Although this Europe-wide and frequently used risk score system for the estimation of early mortality is very valuable, it has some shortcomings. The isolated consideration of age without addressing frailty is one of them. In addition, there are other clinical and prognostic comorbidities, such as liver cirrhosis, and cardiac factors, such as right ventricular function, that can significantly influence mortality and postoperative morbidity.

We believe that other cardiac parameters also contribute to the postoperative outcome. These include, for example, the role of right ventricular function, diastolic dysfunction, left ventricular dilatation in terms of dilated cardiomyopathy or other parameters that serve as an expression of heart failure, such as increased plasma levels of NT-proBNP [15,16,17,18,19].

In this study, we addressed whether, and how, cardiac aging is related to chronological age in cardiac high-risk patients after mitral valve surgery. Furthermore, considering the increasing number of elderly patients requiring surgery, it is essential to identify what the markers of cardiac aging are exactly. It is also necessary to analyze, in more detail, the parameters influencing mortality.

A major dilemma in cardiac surgery relates to elderly high-risk patients. Additionally, complex interventions, such as mitral valve surgery, are associated with high perioperative risks. Such constellations complicate the decision making for or against surgery. 

There is a current trend towards non-surgical treatment of mitral regurgitation using MitraClip to reduce the surgical burdens and complications. However, anatomical, and, thus, physiological, reconstruction is still preferred over the more palliative interventional MitraClip procedure in terms of long-term results. Thus, it is of utmost importance to identify patients who would benefit from anatomical reconstruction and who would also bear the unavoidable surgical burden. 

Thus, in this study we dealt with high-risk patients after mitral valve repair surgery, using our institutional scientific database, which has been in use for almost 20 years.

## 2. Patients and Methods

### 2.1. Ethical Statement

Prior to the start of the study, an ethics vote was obtained from the responsible ethics committee (E-02-20200923, dated 21 November 2020). Patient consent was waived, due to the retrospective study design and complete anonymization of data.

### 2.2. Study Design and Data Collection

The primary objective was to compare cardiac aging between senior and junior patients after cardiac surgery. Secondarily, the senior and junior patients were compared in terms of postsurgical outcome and mortality. Considering the increasing number of seniors requiring surgery, it is also essential to determine the exact markers of cardiac aging.

Furthermore, differences between both age groups in terms of postoperative outcome, in-hospital and 5-year mortality require analysis.

Patients after mitral valve repair were included in the study. The further inclusion criteria were high-risk patient group (EuroSCORE II > 8%) and primary mitral regurgitation as underlying cause of surgery. To minimize confounding variables in the study, we excluded patients with the following baseline conditions from the study: mitral valve endocarditis, ischemic or secondary mitral valve disease, concomitant coronary bypass surgery, patients after mitral valve replacement, and patients with congenital mitral valve disease.

Between 2012 and 2021, a total of 2354 patients underwent mitral valve surgery in our center. After considering the inclusion and exclusion criteria, we identified 1476 patients between 2012 and 2021 after mitral valve repair. There were 133 patients identified who were 80 years or older and 1343 patients who were between 19 and 79 years old.

In this clinical retrospective observational study, patients were divided into 2 age groups. For the senior group the minimum age was set as 80 years, and included patients 80 years and older. All patients between 19 and 79 years were assigned to the junior group.

In order to be able to compare the patients with each other, 1:3 propensity score matching (PS-Matching) was initially performed. Additional parameters were collected from 1476 patients for the purpose of PS matching. Age, and thus the corresponding group assignment, was considered to be the dependent variable. Gender, isolated or concomitant valve surgery, redo surgery, chronic obstructive pulmonary disease, pulmonary hypertension, peripheral arterial disease, arterial hypertension, liver cirrhosis, renal insufficiency, and cardiomyopathy were considered to be independent variables. 

After the PS matching, of the clinically-matched pairs of patients with similar risk profiles, 115, in total, were ≥80 years old, whom we present as Senior in the following, and 345 were <80 years old, whom we refer to as Junior in the following. The PS matching results are shown in Table 1.

To evaluate cardiac aging, the following preoperative transthoracic echocardiographic data were collected: ventricular ejection fraction (LVEF), tricuspid annular plane systolic excursion (TAPSE), diastolic dysfunction, left ventricular end-systolic diameter, left ventricular end-diastolic diameter and left atrial diameter. Moreover, preoperative plasma levels of N-terminal prohormone brain natriuretric peptide (NT-proBNP) were collected. The baseline characteristics of the patients were collected. The postoperative course and complications, as well as in-hospital mortality and 5-year mortality, were also collected and analyzed. For data collection, we used the discharge letter, the surgery report and the examination results from transthoracic echocardiography and laboratory parameters documented in our database.

Furthermore, within the senior and junior groups, we tried to classify patients into cardiac healthy and unhealthy groups, based on left ventricular and right ventricular functions. The intention was to show the influence of cardiac aging related to age on mortality and outcome. Cardiac healthy patients were defined as those with an LVEF ≥ 55% and a TAPSE ≥ 18 mm. The cardiac unhealthy patients, as possible indicators of cardiac aging had an LVEF ≤ 45% and a TAPSE ≤ 15 mm. In the junior group (≤79 years), a total of 125 patients with healthy hearts and 13 patients with unhealthy hearts were found. In the senior group (≥80 years), 31 patients with healthy hearts and 24 patients with unhealthy hearts could be identified.

To further investigate age-related differences in patients, patients were additionally divided into 4 age groups (<60 years, *n* = 65; 60–69 years, *n* = 83; 70–79 years, *n* = 197, and ≥80 years, *n* = 115). Corresponding statistics, including risk profile and postoperative course, as well as mortality, were gathered.

### 2.3. Statistical Analysis

Propensity score (PS) matching and statistical analysis was performed using “R” version 4.1.1 [20]. PS was performed between a group of 80+ years versus <80 years, using the matchit package, for the following factors: gender, isolated vs. concomitant surgery, redo surgery, kidney insufficiency, chronic obstructive pulmonary disease, peripheral arterial disease, arterial hypertension, cardiomyopathy and liver cirrhosis [21]. Numerical variables were compared between both matched groups using Student’s *t*-test for normally distributed data and the Mann–Whitney U test otherwise. Categorical data was compared using Fisher’s exact test and Chi^2^-test, respectively. 

To complement the aforementioned analysis, we also compared four age groups (<60 years, 60–69 years, 70–79 years, ≥80 years), as well as groups reflecting a combination of age (<80, ≥80 years) and cardiac age (low cardiac age: TAPSE ≥ 18 mm, EF ≥ 55%, high cardiac age: TAPSE ≤ 15 mm, EF ≤ 45%). To analyze trends over the four age groups, we used Kendall’s τ correlation for numerical data and the Cochran–Armitage test for trends in proportions for categorical data.

To conduct the comparison between different combinations of cardiac health and age we used ANOVA or the Kruskal–Wallis test for numerical data and the Chi²-test for categorical data. As post hoc tests, we used pairwise Student’s *t*-test or the Mann–Whitney U test with the Holm–Bonferoni correction for numerical data and the Tukey-test for categorical data.

Kaplan Meier survival curves were calculated using the survival package [22].

## 3. Result

### 3.1. Baseline

The senior group had a mean age of 81.60 ± 1.72 years and the junior group a mean age of 67.73 ± 10.19 years. Mean EuroSCORE II was significantly higher in seniors than in juniors (26.53% ± 21.79 vs. 15.51% ± 17.27; *p* < 0.001). Body mass index was significantly lower in seniors (26.3 ± 4.2 vs. 28 ± 5.5 kg/cm^2^, *p* = 0.003).

Pulmonary arterial pressure (PAP) was also significantly higher in seniors than in juniors (43.8 ± 14.1 vs. 38.1 ± 17.6 mmHg, *p* = 0.005). Furthermore, the proportion of preoperatively diagnosed atrial fibrillation was higher in seniors than juniors. However, the difference was not statistically significant (55.4% vs. 47.2%, *p* = 0.166).

Gender, proportion of redo surgery, and comorbidities, such as chronic obstructive pulmonary disease, peripheral arterial disease, arterial hypertension, renal insufficiency, hepatic insufficiency, and cardiomyopathy were comparable in both groups, due to prior PS matching (Table 1).

### 3.2. Cardiac Parameters

To evaluate the cardiac aging, echocardiographic parameters were collected. It could be shown that TAPSE, as an indicator of right ventricular function, was significantly worse in seniors compared to juniors (16.6 mm vs. 21.2 mm, *p* < 0.001). Furthermore, seniors showed significantly larger left ventricular end-systolic diameters and left ventricular end-diastolic diameters (39.3 vs. 33.4 mm, *p* < 0.001; 54.4 vs. 50.8 mm, *p* < 0.001, respectively). Left atrial diameters were also significantly larger in seniors (48.7 vs. 45.6 mm, *p* < 0.001). Left ventricular ejection fraction was slightly lower in the seniors, but the difference was statistically not significant (52% vs. 54.1%, *p* = 0.11). Furthermore, diastolic dysfunction was detected significantly more often in the seniors (*p* < 0.001), Table 2.

Preoperative plasma level of NT-proBNP, as a marker of heart failure, was significantly higher in seniors (10,079 vs. 2727 pg/mL, *p* < 0.001).

Echocardiographic examination of the mitral valve showed the vena contracta to be 7.05 mm in seniors and 7.81 mm in juniors, and the effective regurgitant orifice area (EROA) to be 36.38 in seniors and 42.57 in juniors, while the regurgitation fraction was very similar in both groups (seniors 59%, juniors 59.03%).

### 3.3. Surgical Procedure

In all cases, mitral valve repair was performed, accompanied by a closed semi-rigid annuloplasty ring. In most of the cases, additional repair procedures, such as chordal replacement, quadrangular resection and plication, were performed. All procedures were performed using a cardiopulmonary bypass machine. Mean cardiopulmonary bypass (CPB) time and cross clamp time were 170 and 105 min, respectively, in seniors, and 179 and 115 min, respectively, in juniors. Shorter CPB time and clamping time in seniors was not statistically significant (*p* = 0.234 and *p* = 0.068, respectively).

Combination surgery with aortic or tricuspid valve repair or replacement was performed in 56.52% of seniors and in 57.67% of juniors (Table 3).

Perioperatively, 41 patients received intra-aortic balloon pump (IABP) implantation. IABP implantation was performed in 6.96% of the seniors and 9.57% of the juniors. In addition, an LVAD was implanted in one junior patient.

### 3.4. Postoperative Morbidity and Mortality

The senior patients also showed significantly more severe postoperative courses with higher complication rates. The seniors presented postoperatively significantly more often with respiratory insufficiency (28.9% vs. 11.5%, *p* < 0.001), pneumonia (14% vs. 5.3%, *p* = 0.004), pleural effusion (29.8% vs. 17.7%, *p* = 0.008) and critical illness polyneuropathy and myopathy (CIP/CIM) (7% vs. 0.6%, *p* < 0.001).

The proportion of systemic inflammatory response syndrome and low output syndrome were higher in seniors (29% vs. 9.7%, *p* < 0.001; 11.4% vs. 3.2%, *p* = 0.002, respectively).

Furthermore, postoperative kidney failure and need for dialysis were observed significantly more often in seniors compared to juniors (40.71% vs. 19.12%, *p* < 0.001 and 26.55% vs. 16.13%, *p* = 0.02). 

**Figure 1:**
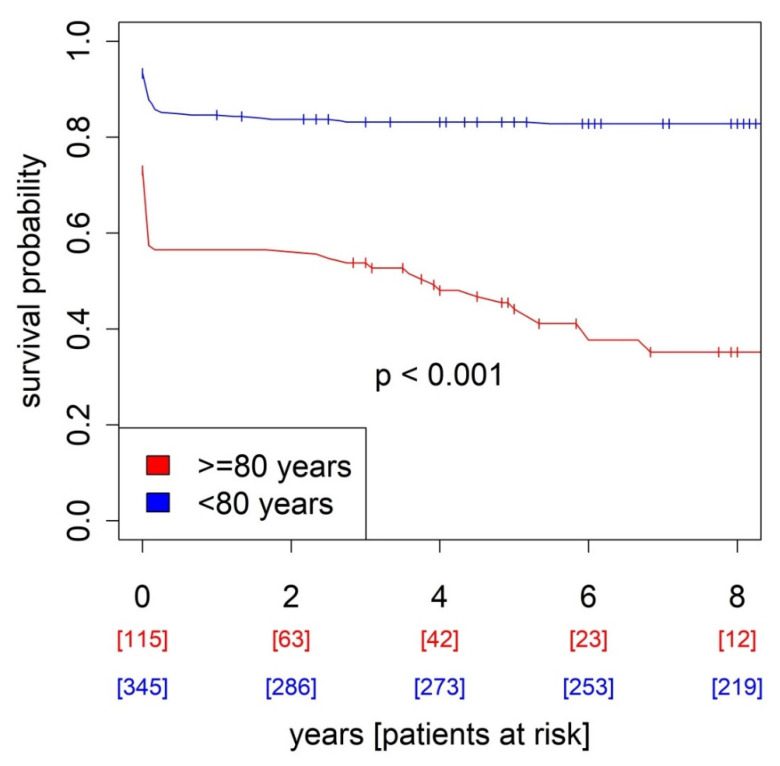
Kaplan–Meier survival curve.

Other complications that occurred more frequently in seniors were observed. However, no statistical significance could be demonstrated. These included stroke (6.31% vs. 3.26%, *p* = 0.256), pericardial tamponade (4.39% vs. 2.64%, *p* = 0.534), bleeding (7.02% vs. 4.4%, *p* = 0.391), and delirium (20.54% vs. 18.34%, *p* = 0.708) (Table 3). The mean duration of hospitalization was 22.7 ± 12.3 days for juniors and 18.5 ± 13.1 days for seniors. In-hospital mortality was 22% for the total cohort. Long-term mortality was 27.2%. Median survival time was 81.38 months. In-hospital mortality was significantly higher in seniors (43.5%) than in juniors (14.8%), *p* < 0.001. Long term, all-cause mortality is shown in the Kaplan–Meier curve (senior: 57.4%, junior: 17.1%, *p* < 0.001) (Figure 1). Mean survival time was 35.38 months for seniors and 96.72 months for juniors.

## 4. Healthy vs. Unhealthy Heart and Its Influence on Mortality and Outcome 

For this purpose, the juniors were grouped into cardiac healthy and unhealthy and the seniors were also grouped into cardiac healthy and unhealthy.

**Figure 2:**
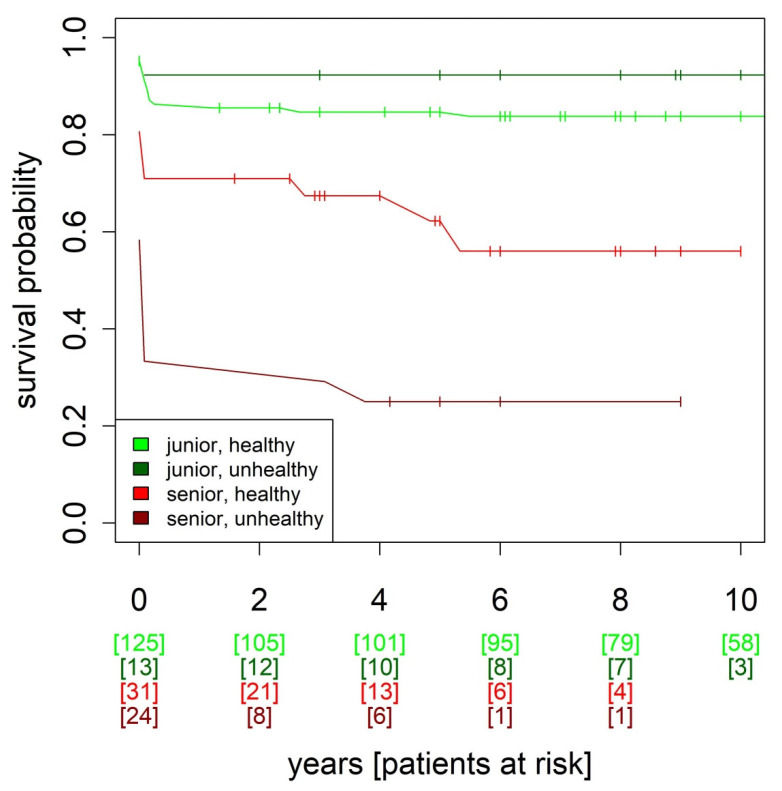
Kaplan–Meier survival analysis of patients of different ages and with different cardiac conditions. Junior < 80 years, senior ≥ 80 years. Healthy = LVEF ≥ 55%, TAPSE ≥ 18 mm, unhealthy = LVEF ≤ 45%, TAPSE ≤ 15 mm.

The analysis between the groups showed significant differences in terms of mortality, acute kidney failure, SIRS, and postoperative atrial fibrillation (*p* < 0.001, respectively), as well as CIP/CIM (*p* = 0.014) and respiratory failure (*p* = 0.002). Subsequent post hoc analysis provided interesting results. While the young cardiac healthy vs. young cardiac unhealthy patients showed no significant differences regarding the abovementioned items, there was a significant difference between young cardiac healthy and old cardiac unhealthy patients in the items, except for respiratory insufficiency and CIP/CIM. Table 4 shows the differences in detail. Figure 2 also shows the survival analysis.

## 5. Preoperative Condition and Postoperative Outcome in Different Life Decades

Regarding the risk profile in different decades of life, some significant differences were shown, including BMI decreasing (*p* < 0.002), EuroSCORE II increasing (*p* < 0.001), and chronic renal failure (*p* = 0.004), as well as NT-proBNP increasing (*p* < 0.001). Most of the echocardiographic parameters also showed significant differences after evaluation of different decades of life, with LAD, TAPSE and diastolic dysfunction (*p* < 0.001) showing significant trends. (Table 5).

**Figure 3:**
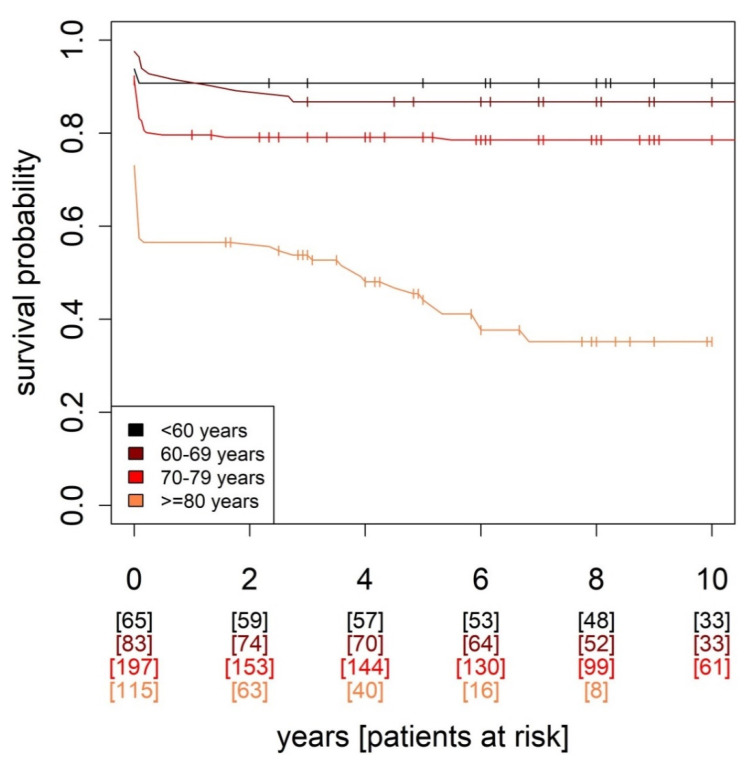
Kaplan–Meier survival analysis in different life decades.

Similarly, we observed, with increasing decades, significant differences in mortality (*p* < 0.001, Figure 3), and in postoperative complications, SIRS, renal failure (*p* < 0.001, respectively), low output syndrome (*p* = 0.009), CIP/CIM (*p* = 0.003), pleural effusion (*p* = 0.003), respiratory insufficiency (*p* < 0.001) and pneumonia (*p* < 0.001) 

## 6. Discussion

A likely reason why hospitalization was longer in younger patients is that the seniors died early and, thus, their overall length of stays seemed shorter. That the overall length of hospital stay was quite long was due to the high-risk patients with extensive severe concomitant diseases.

The analysis of the patients according to age and cardiac aging confirmed the assumption that mortality and postoperative complications inevitably increase with age. However, cardiac aging, in terms of health of the heart, played a relevant role in the postoperative course and elderly patients with a rather healthy cardiac condition had better survival probability than the entire age group (Figure 2 and Figure 3). However, even with a favorable cardiac condition, the detrimental effects of chronological age cannot be completely cancelled out.

The answer to the question, as to why some senior patients have a significantly worse outcome after cardiac surgery than others, has many facets. Comorbidities certainly also play a major role.

However, the impact of cardiac health on outcome is fundamental.

The results of our high-risk cohort suggest that, although perioperative risk increases with age, a wide range of cardiac aging and conditions are present in advanced age, and cardiac condition has a direct effect on outcome. Thus, an elderly patient may not be basically inoperable, nor a young patient always suitable for surgery.

The fact that the elderly cardiac healthy patients have a much better outcome than the cardiac diseased patients can help surgeons and cardiologists in surgical indication. In this study, we used two main cardiac parameters to evaluate cardiac aging. Left ventricular and right ventricular functions represent the most important parameters in the evaluation of cardiac condition. However, we suggest that other cardiac influencing factors may also be important and could be investigated in further studies.

Our data repeated the well-known finding that patients who have reached the eighth decade have a higher mortality risk than younger ones, even with the same risk profiles, as addressed by propensity score matching. They show clear signs of cardiac deterioration, such as significantly larger left ventricular and left atrial diameters, and right ventricular function. Moreover, seniors significantly more often showed diastolic dysfunction and higher plasma levels of NT-proBNP. That LVEF was not significantly worse in the seniors may be a positive selection. Seniors with a relevantly impaired LVEF are usually not considered for cardiac surgery because of higher surgical risks.

The EuroSCORE II (ES II) is a risk score system used in cardiac surgery to estimate the probability of surgical-related mortality. In PS matching, we had to refrain from using the ES II as a variable, because age and LVEF are included in the EuroSCORE II. Significantly higher ES II in seniors may, of course, be partly a result of older age, but certainly also due to higher morbidity.

Another interesting finding in our high-risk cohort was that 14.8% of the juniors with an average ES II of 15.51% died, i.e., the mortality rate here was almost identical to the mortality risk calculation. Of the seniors with a mean ES II of 26.53%, 43.5% died. The fact that mortality in seniors was significantly higher than predicted may have been caused by the underestimation of older age in ES II or by the presence of many other cardiac and noncardiac concomitant diseases at older ages, which are not considered in ES II.

Many postoperative complications, such as kidney failure or CIP/CIM, occur more frequently in seniors, which has already been shown in our previous work [23]. Other studies also confirm our findings that seniors have more atrial fibrillation and suffer from heart failure [24].

Furthermore, not only the recognition of risk factors remains important, but also the treatment or reduction of risk factors. In this field, too, current studies are increasingly being conducted and observed [4,7,25]. 

The number of seniors is estimated to double in the next 25 years and it is assumed that already by 2030 about 20% of the population will be elderly [4]. This suggests the urgent need for proactive development of strategies to manage cardiac aging.

## 7. Conclusions

In summary, seniors are more multimorbid and age is associated with a higher risk of surgery-related mortality. Our results show that seniors with similar underlying cardiac disease suffer significantly more often from certain comorbidities, which can be summarized under the term “cardiac aging”, than juniors. In particular, the all too often neglected parameters for right heart failure and diastolic dysfunction increase the risks contributing to an unfavorable “cardiac age”. Non-cardiac parameters can also be included in “cardiac aging”, such as renal insufficiency, CIP/CIM and SIRS.

Moreover, the postoperative course is more complex and challenging in seniors. Considering the aging society, it is very important to develop proactive strategies for prevention and treatment of cardiac aging. However, the “fountain of youth” is nowhere to be seen. Age alone does predict early outcome. However, for prudent referral of an elderly patient for reconstructive surgery of the mitral valve, with all that this entails, one should consider not only chronological age but also the accumulation of specific comorbidities mentioned above, the so-called “cardiac age”. Fortunately, the term “young at heart” actually exists, literally, and spry seniors without relevant comorbidities affecting the heart and other organs can safely undergo physiological repair of their mitral valves.

Nothing is more counterproductive than an overly liberal decision for surgery in a high-risk patient who would instead be better off with a much less invasive alternative.

## Figures and Tables

**Table 1:** Clinical characteristics of senior and junior patients after propensity score matching.

	Senior (*n* = 115)	Junior (*n* = 345)	* p *
Gender (male)	58.26% (67)	56.81% (196)	0.87
Isolated MVR	56.52% (65)	57.68% (199)	0.913
Redo-Surgery	13.08% (15)	10.72% (37)	0.61
COPD	12.17% (14)	11.88% (41)	1
PAD	6.96% (8)	5.8% (20)	0.822
Cardiomyopathy	5.22% (6)	2.61% (9)	0.289
Liver cirrhosis	0% (0)	0% (0)	1
Hypertension	86.09% (99)	87.25% (301)	0.873
Renal failure	37.39% (43)	35.94% (124)	0.916
Stg. I	17.39% (20)	14.2% (49)	0.497
Stg. II	1.74% (2)	4.93% (17)	0.18
Stg. III	15.65% (18)	13.04% (45)	0.584
Stg. IV	1.74% (2)	1.45% (5)	1
Stg. V	0.87% (1)	2.32% (8)	0.461
No RF	62.61% (72)	64.06% (221)	0.867

MVR: mitral valve repair; COPD: chronic obstructive pulmonary disease; PAD: peripheral arterial disease. Stg: stage; RF: renal failure; Number of seniors after propensity score matching = 115; Number of juniors after propensity score matching = 345.

**Table 2:** Cardiac parameter.

	Senior	Junior	*p*
TAPSE (mm)	16.59 ± 5.5	21.18 ± 5.7	<0.001
LVEF (mm)	52 ± 12.9	54.09 ± 12.7	0.109
LVESD (mm)	39.31 ± 11.1	33.38 ± 8.3	<0.001
LVEDD (mm)	54.37 ± 9.4	50.83 ± 9.1	<0.001
LAD (mm)	48.72 ± 7.2	45.65 ± 8.3	<0.001
Diastolic dysfunction	56.52%	8.41%	<0.001
Grade I	27.83%	6.67%	
Grade II	21.74%	0.87%	
Grade III	6.95%	0.87%	
NT-proBNP (pg/mL)	10,079 ± 11,666	2727 ± 6263	<0.001

TAPSE: tricuspid annular plane systolic excursion; LVEF: left ventricular ejection fraction; LVESD: left ventricular end-systolic diameters; LVEDD: left ventricular end-diastolic diameters; LAD: left atrial diameter; NT-proBNP: N-terminal prohormone brain natriuretric peptide. The data are presented in mean.

**Table 3:** Surgical data and postoperative complications.

	Senior (*n* = 115)	Junior (*n* = 345)	*p*-Value
Surgical data			
CPB time (min)	170.7 ± 63.8	179.8 ± 67.2	0.234
Cross clamp time (min)	105.8 ± 44.1	115.5 ± 45.2	0.068
Concomitant AVR or TVR	56.52%	57.67%	0.913
Postoperative complications			
Respiratory insufficiency	28.9%	11.5%	<0.001
Pneumonia	14%	5.3%	0.004
Pleural effusion	29.8%	17.7%	0.008
CIP/CIM	7%	0.6%	<0.001
Low cardiac output	11.4%	3.2%	0.002
SIRS	29%	9.7%	<0.001
Kidney failure	40.7%	19.1%	<0.001
Dialysis necessity	26.6%	16.1%	0.02
Stroke	6.3%	3.3%	0.256
Pericardial tamponade	4.4%	2.6%	0.534
Delirium	20.5%	18.3%	0.708

CPB: cardiopulmonary bypass; AVR: aortic valve replacement; TVR: tricuspid valve repair; CIP/CIM: critical illness polyneuropathy and myopathy; SIRS: systemic inflammatory response syndrome.

**Table 4:** Outcome differences between cardiac healthy versus cardiac unhealthy in juniors versus seniors.

	Junior, Cardiac Healthy	Junior, Cardiac Unhealthy	Senior, Cardiac Healthy	Senior, Cardiac Unhealthy	* p *	
LVEF	61.62 ± 5.1	31.69 ± 9.2	61.52 ± 4.65	34.33 ± 7.98	<0.001	
TAPSE	23.58 ± 4.04	11.77 ± 3.96	23.1 ± 3.76	12 ± 1.89	<0.001	
Diastolic dysfunction	2.4%	38.46%	25.81%	91.67%	<0.001	
Postoperative outcome						
In-Hospital Mortality	11.38%	7.69%	29.03%	66.67%	<0.001	
SIRS	6.61%	15.38%	22.58%	50%	<0.001	
CIP/CIM	0%	0%	6.45%	8.33%	0.014	
Acute kidney failure	13.93%	23.08%	32.26%	54.17%	<0.001	
Atrial fibrillation	7.32%	0%	29.03%	41.67%	<0.001	
Respiratory insufficiency	8.33%	30.77%	29.03%	25%	0.002	
* p * -value after posthoc analysis						
	Ju healthy vs. Ju unhealthy	Ju healthy vs. Se healthy	Ju healthy vs. Se unhealthy	Ju unhealthy vs. Se healthy	Ju unhealthy vs. Se unhealthy	Se healthy vs. Se. unhealthy
In-Hospital Mortality	1	0.087	<0.001	0.503	0.01	0.049
SIRS	1	0.101	<0.001	1	0.266	0.266
CIP/CIM	-	0.215	0.126	1	1	1
Acute kidney failure	1	0.168	<0.001	1	0.561	0.561
Atrial fibrillation	0.979	0.011	<0.001	0.231	0.078	0.979
Respiratory insufficiency	0.213	0.031	0.213	1	1	1

LVEF: left ventricular ejection fraction; TAPSE: tricuspid annular plane systolic excursion; SIRS: systemic inflammatory response syndrome; CIP/CIM: critical illness polyneuropathy and myopathy; Ju: Junior; Se: Senior; The data are presented in mean or %.

**Table 5:** Risk profiles and postoperative complications of four different decades.

	<60 Years (*n* = 65)	60–69 Years (*n* = 83)	70–79 Years (*n* = 197)	≥80 Years (*n* = 115)	* p *
Risk profile					
BMI (kg/cm^2^)	28.68 ± 6.46	28 ± 4.8	27.73 ± 5.37	26.29 ± 4.15	0.002
EuroSCORE II (%)	10.31 ± 17.23	11.95 ± 13.81	18.7 ± 18.01	26.53 ± 21.88	<0.001
Kidney failure	21.54%	26.51%	44.67%	37.39%	0.004
Concomitant surgery	43.08%	55.42%	63.45%	56.52%	0.063
NT-proBNP (pg/mL)	1998 ± 2927	3008 ± 7022	2799 ± 6566	10079 ± 11,666	<0.001
Echocardiographic data					
LVESD (mm)	35.2 ± 8.91	34.73 ± 7.88	32.22 ± 8.1	39.31 ± 11.12	0.057
LVEDD (mm)	52.37 ± 10.25	54.04± 8.59	48.97 ± 8.5	54.37 ± 9.39	0.563
LAD (mm)	42.46 ± 7.14	47.57 ± 7.69	45.89 ± 8.57	48.72 ± 7.18	<0.001
LVEF (%)	53.11 ± 12.77	54.83 ± 13.49	54.1 ± 12.3	52 ± 12.93	0.362
TAPSE (mm)					<0.001
Diastolic dysfunction	6.15%	9.64%	8.63%	57.39%	<0.001
Postoperative outcome					
In-Hospital Mortality	9.23	6.02%	18.56%	41.47%	<0.001
SIRS	6.15%	7.41%	11.79%	28.95%	<0.001
Low output syndrome	3.08%	2.47%	3.59%	11.4%	0.009
Pleural effusion	10.94%	18.52%	19.49%	29.82%	0.003
CIP/CIM	0%	1.23%	0.51%	7.02%	0.003
Acute kidney failure	12.5%	9.88%	25.13%	40.71%	<0.001
Atrial fibrillation	16.92%	7.41%	9.28%	21.93%	0.164
Pneumonia	1.54%	2.47%	7.69%	14.04%	<0.001
Respiratory insufficiency	7.81%	12.35%	12.31%	28.95%	<0.001

BMI: body mass index; NT-proBNP: N-terminal prohormone brain natriuretric peptide; LVESD: left ventricular end-systolic diameters; LVEDD: left ventricular end-diastolic diameters; LAD: left atrial diameter; LVEF: left ventricular ejection fraction; TAPSE: tricuspid annular plane systolic excursion; SIRS: systemic inflammatory response syndrome; CIP/CIM: critical illness polyneuropathy and myopathy. The data are presented in mean or %.

## Data Availability

Data will not be published for privacy reasons and will be saved at the clinic.
